# The contribution of hypothalamic macroglia to the regulation of energy homeostasis

**DOI:** 10.3389/fnsys.2014.00212

**Published:** 2014-10-22

**Authors:** Laura B. Buckman, Kate L. J. Ellacott

**Affiliations:** ^1^Department of Molecular Physiology and Biophysics, Vanderbilt University Medical CenterNashville, TN, USA; ^2^Division of Infectious Disease, School of Medicine, University of North Carolina at Chapel HillChapel Hill, NC, USA; ^3^Biomedical Neuroscience Research Group, University of Exeter Medical School, Hatherly LaboratoriesExeter, UK

**Keywords:** astrocyte, tanycyte, fasting, obesity, high-fat diet, inflammation, hypothalamus

## Abstract

The hypothalamus is critical for the regulation of energy homeostasis. Genetic and pharmacologic studies have identified a number of key hypothalamic neuronal circuits that integrate signals controlling food intake and energy expenditure. Recently, studies have begun to emerge demonstrating a role for non-neuronal cell types in the regulation of energy homeostasis. In particular the potential importance of different glial cell types is increasingly being recognized. A number of studies have described changes in the activity of hypothalamic macroglia (principally astrocytes and tanycytes) in response to states of positive and negative energy balance, such as obesity and fasting. This article will review these studies and discuss how these findings are changing our understanding of the cellular mechanisms by which energy homeostasis is regulated.

## Energy homeostasis

Energy homeostasis, the balance between food intake and energy expenditure, is an exquisitely sensitive process regulated by the central nervous system (CNS). Mediated by nutritional, humoral and nervous inputs into the brain from the periphery, information on energy status is integrated by hypothalamic and brainstem nuclei, and relayed to other neural circuits such as those of the mesolimbic dopaminergic pathway. To date the vast majority of research on the hypothalamic regulation of energy homeostasis has focused on the involvement of neuronal signaling with the contribution of other cell types only beginning to be addressed (Yi et al., 2011; Chowen et al., 2013). This mini-review will summarize and discuss the emerging evidence for a role of one of these cellular classes, hypothalamic macroglia, in the regulation of energy homeostasis focusing on states of both positive (e.g., obesity and neonatal overnutrition) and negative energy balance (e.g., fasting).

## Macroglia

In the adult CNS glia are broadly categorized into two main classes: (1) macroglia—including astrocytes, oligodendrocytes, NG-2 glia, tanycytes and other ependymal cells; and (2) microglia. Originally thought to be largely structural cells acting as “glue” in the CNS it is now widely accepted that glia are critical regulators of parenchymal homeostasis and directly influence neuronal communication (Tasker et al., 2012). As will be discussed in more detail below, changes in hypothalamic glial cell activity have been reported in states of positive and negative energy balance in rodents (Hsuchou et al., 2009a; Horvath et al., 2010; García-Cáceres et al., 2011; Thaler et al., 2012; Buckman et al., 2013; Langlet et al., 2013) and non-human primates (Grayson et al., 2010), suggestive of an active role in the regulation of energy homeostasis. Furthermore, consistent with data from animal models, evidence of gliosis, a condition associated with increased numbers of activated glia, has been discovered in humans with elevated body mass index using neuroimaging techniques (Drake et al., 2011; Thaler et al., 2012). Due to space constraints this mini-review is focused on the contribution of macroglia, primarily astrocytes and tanycytes, to the regulation of energy homeostasis; however, it is not our intention to underplay the importance of microglia to this area which is likely to be highly significant (Bilbo and Schwarz, 2012).

### Astrocytes

Astrocytes are macroglial cells of ectodermal origin. In the human brain, astrocytes constitute nearly half of the cells in the CNS, in some brain regions outnumbering neurons by a factor of ten to one. The functions of astrocytes in the CNS are diverse (extensively reviewed by Abbott et al., 2006; Sofroniew and Vinters, 2010; Tasker et al., 2012) and include: (1) modulating neuronal activity; (2) energy storage and supply to neurons; and (3) regulation of blood-brain barrier (BBB) permeability and cerebral blood flow. Astrocytes play a critical role in regulating other neuroendocrine processes in the hypothalamus including activity of gonadotrophin-releasing hormone (Prevot et al., 2007) and magnocellular (Panatier, 2009; Tasker et al., 2012) neurons. Intriguingly, hypothalamic astrocytes also show diurnal variation in their morphology and gene expression (reviewed in Jackson, 2011) and play a critical role in the control of circadian rhythms (Ng et al., 2011).

### Oligodendrocytes and NG-2 glia

In common with astrocytes, oligodendrocytes are derived from the ectoderm; however, their principal function is believed to be the formation of myelin which insulates neuronal axons, facilitating salutatory conduction. A third type of macroglia, NG-2 glia, are more recently described, and as such are less well characterized. NG-2 glia act as oligodendrocyte precursors but also have functional similarities to astrocytes (Nishiyama et al., 2005). To date there is minimal published evidence for a role for oligodendrocytes or NG-2 glia in the regulation of energy homeostasis. However, given the recent focus on the functional role of astrocytes in the modulation of energy homeostasis and the potential functional overlap between NG-2 glia and astrocytes, it is likely that time will reveal that these cells also contribute to the regulation of neuroendocrine circuitry. Indeed, recent evidence suggests that NG-2 glia are capable of differentiating into neurons within the hypothalamus (Robins et al., 2013b). Furthermore, specific deletion of the NG-2 proteoglycan from oligodendrocytes results in a lean phenotype in mice (Chang et al., 2012), but whether this occurs due to specific alterations in oligodendrocyte action or as a result of a global deficit in neuronal function as a consequence of hypomyelination is not known.

### Ependymal cells/Tanycytes

Ependymal cells are dedicated glia lining the cerebral ventricles. Tight-junctions between cells of the ependymal layer regulate the exchange of substances between the cerebral-spinal fluid and CNS parenchyma. Within the third and fourth ventricles there is a specialist population of ependymal cells called tanycytes which are divided into four subclasses (α1, α2, β1 and β2) distinguished by their morphology and localization (for review see Rodríguez et al., 2005). In the hypothalamus a subpopulation of α-tanycytes is believed to have the capacity to act as neural stem cells in the adult brain (Robins et al., 2013a). The reader is referred to a comprehensive review by Bolborea and Dale published in 2013 on the potential role of hypothalamic tanycytes in the regulation of food intake and energy homeostasis (Bolborea and Dale, 2013). In light of this we have tried to focus our discussion of tanycytes to pertinent work that has been published subsequent to their review article.

## Macroglial changes in states of positive energy balance

### Astrocytes are responsive to high-fat feeding, obesity and neonatal overnutrition

Diet-induced obesity causes activation of hypothalamic inflammatory signaling pathways (De Souza et al., 2005). In part due to their intimate relationship with neurons and the BBB, astrocytes have fundamental roles in the innate immune response of the CNS and undergo key morphological changes in CNS pathologies. In the context of inflammation, astrocytes respond by a process referred to as reactive astrogliosis, characterized by cellular hypertrophy, proliferation, and increased expression of the intermediate filaments: glial-fibrillary acidic protein (GFAP), vimentin, and nestin (Sofroniew, 2009). Reactive astrogliosis is a graded phenomenon, ranging from mild alterations in gene expression and cellular morphology to severe (sometimes permanent) tissue reorganization leading to glial scars (Sofroniew, 2009). Changes in astrocyte morphology indicative of reactive astrogliosis have been reported in rodent models of chronic-diet induced obesity (Hsuchou et al., 2009a; Horvath et al., 2010; Thaler et al., 2012; Buckman et al., 2013), genetic obesity (Hsuchou et al., 2009a; Buckman et al., 2013) and neonatal overnutrition (García-Cáceres et al., 2011; Fuente-Martín et al., 2012). In adult animals obesity-associated reactive astrogliosis has been implicated in altering the firing of neurons of the central melanocortin system (Horvath et al., 2010); thus, potentially contributing to the perpetuation of obesity by modulating the tone of this neuronal circuit critical for the regulation of energy homeostasis (Cone, 2005). However, further work is required to determine the precise molecular mechanism(s) by which astrocytes influence neuronal activity in the context of obesity. The extensive structural changes in hypothalamic astrocytes seen during obesity and neonatal overnutrition suggests that these cells may directly contribute to synaptic rewiring physically by changing glial ensheathment of neurons and also by altering the ability of neurons to receive direct humoral inputs from the periphery (Horvath et al., 2010). Additionally, astrocytes might directly modulate neuronal activity during obesity *via* the action of glial-derived factors, commonly referred to as gliotransmitters, or the modulation of glutamate availability in the synaptic cleft. The presence of astrogliosis in a rodent model of neonatal overnutrition (García-Cáceres et al., 2011; Fuente-Martín et al., 2012) likely has significant implications for the developmental wiring of hypothalamic feeding circuitry, which influences the propensity of these animals to go on to develop obesity in adulthood.

Recent immunohistochemical data suggests that obesity-associated morphologic changes in glial cells of adult rodents are reversible following weight-loss (Berkseth et al., 2014); however, it remains to be fully determined how obesity functionally alters glial-cell activity and whether these changes are restored following weight-loss.

In addition to modulation by chronic high-fat feeding and neonatal overnutrition, changes in markers of astrocyte activation have been reported as early as twenty-four hours after introduction of a high-fat diet to rodents (Thaler et al., 2012). This suggests that astrocytes may also be involved in modulating the acute homeostatic response to high-fat feeding. Indeed, recent work by Le Foll et al. suggests that ketone bodies produced by astrocytes of the ventromedial hypothalamus contribute to the regulation of acute high-fat chow intake in a restricted feeding paradigm (Le Foll et al., 2013). This indicates that astrocytes directly contribute to the regulation of food intake in response to acute high-fat feeding in part by modulating energy availability to key hypothalamic neurons.

The molecular trigger(s) for astrocyte activation/astrogliosis in response to high-fat feeding and obesity are still unclear. It is intriguing that obesity associated-astrogliosis does not occur uniformly across the hypothalamus (Buckman et al., 2013) suggesting that regional factors such as proximity to the third ventricle, BBB permeability, neuronal activity and neurochemical identity may factor in the response. The inflammatory constituents in obesogenic diets are unlikely to be the sole stimuli for obesity-associated astrogliosis. The finding that in addition to diet-induced obesity, hypothalamic astrogliosis is present in rodent models of genetic obesity (Hsuchou et al., 2009a; Buckman et al., 2013) when the animals are maintained on standard laboratory chow, suggests that other humoral and molecular changes associated with obesity contribute to this response. The potential importance of humoral signaling in glial cell activation is further supported by studies in microglia (Gao et al., 2014). Data from *in vitro* studies indicate that saturated fatty acids activate inflammatory signaling in primary astrocyte cultures (Gupta et al., 2012); however, other potential triggers for obesity-associated astrogliosis include: metabolic endotoxemia as a result of changes in the gut microbiome (Cani et al., 2007); endoplasmic reticulum stress (Zhang et al., 2008); neuronal injury (Thaler et al., 2012) and potentially neurogenic neuroinflammation arising from increased neuronal activity (Xanthos and Sandkühler, 2014). Further work is required elucidate the molecular triggers for obesity-associated astrogliosis; however, it is likely to be a highly complex process involving the integration of multiple signals and cues.

### Macroglia are directly responsive to key metabolic hormones

Astrocytes can directly respond to humoral stimuli further supporting the hypothesis that these cells contribute to the regulation of energy homeostasis under normal physiologic conditions as well as during pathologic states such as obesity. For example, astrocytes express receptors for the key adipostatic hormone leptin (Hsuchou et al., 2009a,b; Kim et al., 2014) suggesting that astrocytes may be capable of directly responding to changes in adipose tissue energy stores by modulating the activity of hypothalamic neuronal circuitry. Indeed, experimental evidence indicates that chronic intracerebroventricular leptin treatment regulates the expression of both structural astrocyte proteins, vimentin and GFAP (García-Cáceres et al., 2011), and key glutamate and glucose transporters on hypothalamic astrocytes (Fuente-Martín et al., 2012). Furthermore, mice with germ-line deletion of leptin-receptors from astrocytes show a modest protection from diet-induced obesity (Jayaram et al., 2013); however, in this study it was not determined whether this was the result of a failure of the astrocytes to become reactive in response to high-fat feeding or due to other alterations in the astrocyte activity.

A more recent study utilizing a mouse model with tamoxifen-inducible deletion of leptin receptors from astrocytes of adult mice indicates that intact leptin signaling in astrocytes important for the actions of this hormone in the regulation of energy homeostasis (Kim et al., 2014). For example, it was demonstrated in this study that the ability of leptin to reduce food intake is partially inhibited following was loss of astrocytic leptin-receptor signaling in adult mice. Furthermore, mice with loss of astrocytic leptin signaling show reduced astrocytic coverage of ARC melanocortin neurons leading to altered synaptic inputs to these neuronal populations that are critical for the regulation of feeding behavior.

In addition to leptin receptors, astrocytes have also been reported to express insulin (Albrecht et al., 1982) and melanocortin-4 receptors (Selkirk et al., 2007) *in vitro* in primary culture. Furthermore, in astrocytes insulin has been shown to directly regulate cell surface expression of the glutamate transporter, excitatory amino acid transporter 2 (EAAT2/GLT1; Ji et al., 2011) providing a potential humoral mechanism for the astrocytic-regulation of synaptic activity during obesity.

Emerging evidence suggests that in common with neurons, macroglia develop hormone resistance during chronic obesity. A recent study by Balland and colleagues demonstrates that median eminence tanycytes of diet-induced obese mice show reduced STAT3 phosphorylation in response to leptin treatment (Balland et al., 2014). The investigators also go on to show that intact leptin signaling in tanycytes is required for leptin transport into the medial basal hypothalamus, which may represent a new regulatory node for central hormone sensitivity. Moreover, it is likely that astrocytes are also capable of developing leptin and/or insulin resistance; however direct *in vivo* evidence of astrocytic hormone resistance during obesity is lacking.

### The neurogenic capacity of tanycytes is regulated by high-fat feeding

In 2005 Kokoeva et al. first reported neurogenesis of hypothalamic neurons in adult mice, suggestive of a role for these cells in the regulation of energy homeostasis (Kokoeva et al., 2005). In a subsequent study the same group demonstrated that turn-over of hypothalamic neurons was suppressed in diet-induced obese mice, linked to a reduction in neurogenesis and an increase in apoptosis of the newly divided cells (McNay et al., 2012). Activation of the inflammatory Iκκβ/NFκB pathway in neural stem cells *in vitro* and *in vivo* reduces their neurogenic capacity and subsequent survival (Li et al., 2012) providing a novel mechanistic link between CNS inflammation and the regulation of energy homeostasis. Recent work shows that tanycytes are key components of this neurogenic niche (Lee et al., 2012; Robins et al., 2013a) suggestive of a unique role for these macroglia in the regulation of energy homeostasis.

## Macroglial changes in states of negative energy balance

### Astrocytes are critical mediators of the response to hypoglycemia

The energy needs of the brain are prioritized at all costs during states of low energy availability. Indeed, astrocytic glycogen stores are critical to sustain neurons under these conditions (Suh et al., 2007). Thus, due to their roles in glucose uptake into the CNS and in providing energy to neurons it is logical that macroglia must be superbly sensitive to glycemic state, and this is supported by published data. For example, pharmacologic inhibition of metabolic activity in hypothalamic glia reduces neuronal activation in response 2-deoxyglucose induced glucoprivation (Young et al., 2000) establishing that glia are critical for the neuronal response to low glucose. Subsequently, expression of the glucose transporter GLUT-2 in astrocytes was discovered to be vital for CNS sensing of hypoglycemia when it was shown that overexpression of GLUT-2 in astrocytes was sufficient to restore hypoglycemia-induced peripheral glucagon release in animals with GLUT-2 deficiency (*Ripglut1;glut2−/–* mice; Marty et al., 2005). Further studies also implicate the type 1 glucose transporter (GLUT-1) in hypothalamic astrocytes in sensing changes in peripheral glucose homeostasis (Chari et al., 2011).

Recent data suggests that in addition to astrocyte-neuronal interactions, intact astrocyte-astrocyte networks in the medial-basal hypothalamus are also important for glucose sensing. Allard and colleagues demonstrated that reducing cell-cell transfer of small molecules *via* gap-junctions in hypothalamic astroglial networks *via* inhibition of connexin-43, results in reduced hypothalamic glucose sensitivity (Allard et al., 2014). Further studies in this area are necessary to fully elucidate the molecular mechanisms by which astrocytes communicate information on glycemic status to neurons. Is it due to astrocyte mediated changes in substrate availability as suggested by the work of Arrieta-Cruz et al. (2013)? Alternatively, are there also direct regulatory roles for astrocytes in modulating the activity of glucose sensing neurons in the hypothalamus *via* the release of gliotransmitters, the modulation of neurotransmitter reuptake, and/or alterations in synaptic plasticity?

### Tanycytes are directly responsive to fasting

The localization of tanycytes at the base of the third ventricle, proximal to the median eminence, makes them ideally positioned to act as gatekeepers between the periphery and the brain *via* the cerebroventricular system. Tanycytes are glucose sensitive (for review see Bolborea and Dale, 2013) and respond to reduced circulating glucose levels associated with fasting by altering the permeability of their tight-junctions which subsequently increases access of circulating factors to the CNS (Langlet et al., 2013).

In addition to a role in altering permeability of the blood-hypothalamus barrier in response to fasting, there is also evidence that tanycytes modulate energy homeostasis *via* their neurogenic capacity. Median eminence Fgf10-expressing tanycytes (thought to be largely of the β-subtype) have been demonstrated to repopulate the arcuate nucleus of the hypothalamus in the fasted state where they become NPY expressing neurons (Haan et al., 2013); thus, promoting a rise in food intake by increasing orexigenic drive.

## Evidence for a role of gliotransmitters in regulation of food intake

Endozepines are endogenous proteins with “benzodiazepine-like” binding capacity and include the diazepam-binding inhibitor (DBI) and its cleavage products including the octadecaneuropeptide (ODN; DBI_33-50_). Expressed at high levels in hypothalamic macroglia (astrocytes and tanycytes; Tonon et al., 1990), endozepine expression is down-regulated by fasting in mice and rats (Compère et al., 2010; Lanfray et al., 2013), providing evidence of metabolic control of this system. The finding that CNS administration of ODN significantly reduces food intake in rodents further supports a potential role in the regulation of energy homeostasis (de Mateos-Verchere et al., 2001; Compère et al., 2003; Lanfray et al., 2013). While the mechanisms remain to be fully elucidated, there is some data to suggest that the anorexigenic actions of endozepines are not mediated *via* a classical benzodiazepine receptor but *via* a metabotropic receptor (do Rego et al., 2007) and also require intact central melanocortin-3/4 receptor signaling (Lanfray et al., 2013). Moreover, as astrocytic DBI is known to potentiate inhibitory GABAergic transmission (Christian and Huguenard, 2013) it is likely that modulation of this important neurotransmitter pathway may contribute to the anorexigenic actions of this peptide. In addition to binding the GABA_A_ receptor, endozepines also show high binding affinity for the mitochondrial translocator protein (TSPO). Intriguing, in the CNS TSPO is largely localized within glia and is highly upregulated by glial cell activation leading to the widespread use of TSPO ligands as *in vivo* neuroimaging agents for CNS pathologies (Venneti et al., 2013). This indicates that another potential mechanism for the anorexigenic activity of endozepines may be *via* direct modulation of glial cell activity. Further studies, including examining the phenotype of animal models with alterations in endozepine signaling are necessary to clarify the physiologic role for this pathway in the regulation of energy homeostasis.

## Summary

It is apparent from the wealth of emerging evidence that, in addition to their role in other neuroendocrine processes, hypothalamic macroglia have a critical role in the regulation of energy homeostasis. While much of the current research is focused on macroglial-neuronal interactions in the regulation of energy homeostasis, it is highly likely that the interaction between glial cell types, such as microglia and astrocytes, also represents a key regulatory node. Important work remains to be done to determine the roles of hypothalamic macroglia in the physiologic maintenance of energy balance as well as their contribution to disease pathology when an organism is out of homeostasis in disease states such as obesity and cachexia.

## Conflict of interest statement

The authors declare that the research was conducted in the absence of any commercial or financial relationships that could be construed as a potential conflict of interest.
